# The relationship between potential categories of self-management behavior and disease knowledge level of gout patients based on K-means clustering analysis

**DOI:** 10.3389/fpsyg.2026.1951213

**Published:** 2026-10-09

**Authors:** Zuoyun Ma, Xia Pan, Tianwang Li, Yali Wang, Chun Zheng, Li Tang

**Affiliations:** 1 School of Nursing, Guangdong Pharmaceutical University, Guangzhou, Guangdong, China; 2 Department of Rheumatology and Immunology, The Affiliated Guangdong Second Provincial General Hospital of Jinan University, Guangzhou, Guangdong, China; 3 Department of Nursing, The Affiliated Guangdong Second Provincial General Hospital of Jinan University, Guangzhou, Guangdong, China

**Keywords:** cluster analysis, disease knowledge level, gout, patient subtypes, self-management behavior

## Abstract

**Objective:**

To explore the potential categories of self-management behaviors of gout patients, analyze the relationship between each category and the disease knowledge level, and provide a basis for formulating targeted intervention strategies.

**Methods:**

A total of 402 patients with gout were selected by convenience sampling. The general information questionnaire, the gout patients’ self-management assessment scale, and the knowledge level measurement questionnaire were used in this study. K-means cluster analysis was used to identify the potential categories of self-management behavior.

**Results:**

The score of self-management behavior of gout patients was (142.30 ± 29.53), which could be divided into four categories: high self-management-knowledge adequacy type (28.6%), low self-management-knowledge deficiency type (15.4%), moderate self-management-insufficient transformation type (30.8%), and good self-management-knowledge deficiency type (25.1%). There were statistically significant differences in age, residence, number of acute flares in the past year, serum uric acid (SUA) level and disease knowledge level among different categories (*p* < 0.05).

**Conclusion:**

There is heterogeneity in self-management behavior of gout patients, and there are differences in disease knowledge level among different types of patients. Medical staff should implement hierarchical and precise intervention based on category characteristics.

## Introduction

Gout is a common inflammatory joint disease caused by the deposition of monosodium urate crystals in joints and surrounding tissues (Dalbeth et al., 2021). The global prevalence and incidence of gout continue to rise (Dehlin et al., 2020), with more than 9.4 million new cases and nearly 57 million patients (Jin et al., 2025). Gout has become an important public health challenge. Although the treatment of lowering uric acid has been relatively mature, the rates of treatment, compliance, and awareness of the disease are still at a low level (Son et al., 2021), suggesting that it is difficult to achieve ideal disease control by relying solely on drug treatment, and the patient’s self-management behavior plays an irreplaceable role in the long-term management of gout.

Despite the availability of effective urate-lowering therapy, many patients still have suboptimal treatment adherence and insufficient disease knowledge (Derksen et al., 2017), with significant individual differences in self-management behaviors. Gout patients face prominent problems such as diverse sources of disease knowledge and uneven quality, lack of attention to asymptomatic management, and difficulty in changing long-term bad living habits, which seriously restrict the effective implementation of self-management behavior (Hao et al., 2024). At present, there are great individual differences in self-management behaviors of gout patients. In-depth understanding of the characteristics associated with these differences is the premise of implementing stratified and precise intervention. As the medical model has shifted from “disease-centered” to “patient-centered,” the role of patients’ cognitive level of disease in behavioral change has received increasing attention (Yang et al., 2026). However, most of the existing studies regard disease knowledge as a single continuous variable, ignoring the possible heterogeneous structure of disease knowledge within the population, and it is difficult to effectively guide stratified intervention.

Compared with traditional methods, K-means clustering can divide individuals into different subgroups according to their characteristics in multiple dimensions, effectively revealing the heterogeneity within the group (Dunn et al., 2018; Kim et al., 2026). Based on this, this study intends to use K-means clustering analysis method to identify the potential categories of self-management behaviors of gout patients, and to explore the differences in disease knowledge levels among different categories and their associated factors, in order to provide basis and practical guidance for individualized health management of gout patients.

## Materials and methods

### Research design

This study used a cross-sectional survey design.

### Research object

Convenience sampling method was used to select gout patients from the Department of Rheumatology and Immunology of a tertiary hospital in Guangdong Province from October 2025 to June 2026 as the research object. Inclusion criteria: (1) meeting the 2015 ACR/EULAR classification criteria (Neogi et al., 2015); (2) diagnosed gout ≥ 6 months; (3) Age ≥ 18 years old; (4) Normal reading and writing ability, good language communication ability; (5) Informed consent and voluntary participation in this study. Exclusion criteria: (1) with malignant tumor, or with severe heart, liver and kidney dysfunction; (2) Mental disorders, cognitive dysfunction.

Based on the Kendall sample size estimation method (Ni et al., 2010), that is, the sample size is 5 to 10 times the variable. The number of variables included in this study was 15, and the 10% loss rate was considered. The sample size should be 84–167 cases, and 402 cases were finally included.

### Research tools

#### General information questionnaire

Self-designed by the research group, including gender, age, BMI, residence, work status, education level, family per capita monthly income, course of disease, family history, tophi, current disease stage, number of acute flares in the past year, and the latest serum uric acid (SUA) level.

#### Gout patients self-management assessment scale

The scale was compiled by Yao et al. (2020), including 4 dimensions and 41 items. The four dimensions are: disease treatment management, diet management, lifestyle management, and psychosocial management. The Likert 5-level scoring method was used to score 1–5 points respectively, with a total score of 41–205 points. The higher the score, the better the patient’s self-management behavior. The Cronbach’s α coefficient of the scale was 0.962. In this study, the Cronbach’s α coefficient for the total scale was 0.943, and the Cronbach’s α coefficients for the four subdimensions were: 0.801 for disease treatment management, 0.743 for dietary management, 0.792 for lifestyle management, and 0.819 for psychosocial management, indicating good internal consistency.

#### Gout patients’ knowledge level measurement questionnaire

The questionnaire was developed by Zhang et al. (2011) and included 10 questions: 2 on the pathogenesis of gout, 2 on the symptoms and treatment of acute gout attacks, 5 on the management and prevention of chronic gout, and 1 on the complications of gout. The correct answer of each item was 1 point, the total score was 0–10 points, and the score ≥ 7 points (the median value of the original validation sample) was considered that the patient knew the knowledge of gout. The Flesch–Kincaid readability score of this questionnaire was 4.7 and the Flesch readability was 81.4%.

### Data collection methods

The researchers introduced the research purpose to the research object. After obtaining the informed consent, the questionnaire was distributed on the spot to guide the actual answer. On-site recovery and integrity verification, timely verification and confirmation of suspicious items. A total of 410 questionnaires were distributed, and 402 valid questionnaires were recovered, with an effective recovery rate of 92.84%.

### Statistical analysis

Statistical analysis was performed using IBM SPSS27.0 software. In order to identify the potential categories of self-management behavior of gout patients, this study used the scores of four dimensions of self-management assessment scale of gout patients as clustering variables. Due to the different number of items in each dimension, the total score range of each dimension is different. In order to eliminate the dimensional differences and ensure the equal contribution of each dimension to the clustering solution, the original scores of each dimension are standardized into *Z*-score values before clustering. The elbow method, the downward trend of the sum of squares (WSS), iterative convergence and clinical interpretability were used to determine the optimal number of clusters. After determining the optimal clustering scheme, chi-square test and Kruskal–Wallis *H* test were used for single factor analysis to compare the differences in demography, clinical characteristics and disease knowledge among different potential categories of patients. Variables with *p* < 0.05 in univariate analysis were included in multivariate logistic regression to identify factors associated with potential categories. The goodness of fit of the model was evaluated by likelihood ratio test and pseudo-*R*^2^ index. Multicollinearity was evaluated by variance inflation factor (VIF). Bilateral *p* < 0.05 was considered statistically significant. In addition, in order to verify the robustness of the clustering scheme, this study uses different random initial centers to repeat K-means clustering three times, and randomly selects 70% of the samples for repeated clustering to evaluate the consistency of the clustering results.

## Results

### General information and scale scores of gout patients

A total of 402 patients were included in this study. The score of self-management assessment scale for gout patients was (142.30 ± 29.53) points, among which the score of disease treatment management was (47.04 ± 10.79) points, the score of diet management was (36.71 ± 9.11) points, the score of lifestyle management was (37.69 ± 9.61) points, and the score of psychosocial management was (20.88 ± 5.89) points. The knowledge level score of gout patients was (5.71 ± 1.97), and the awareness rate was 35.1%.

### Cluster analysis of self-management behavior of gout patients

In this study, the elbow method was used to preliminarily determine the optimal number of clusters (Kristianto et al., 2026). The results showed that (see Figure 1), the sum of squares (SSE) in the group gradually decreased with the increase of the number of clusters. When k increases from 3 to 4, the decrease of SSE decreases from 195.7 to 85.3, a decrease of 110.4; when k increases from 4 to 5, the decrease of SSE is 70.2, and the downward trend has obviously tended to be gentle, suggesting that the inflection point is located at k = 4. Further combined with the iterative convergence test, it is found that the clustering centers of the three and five types of schemes are still not completely stable after reaching the maximum number of iterations (10 times) (the maximum center changes are 0.011 and 0.034, respectively), indicating that the structural stability of these two schemes is insufficient. After 10 iterations, the change of cluster center of the four schemes is reduced to 0.000, which shows that the scheme has good convergence stability.

**Figure 1:**
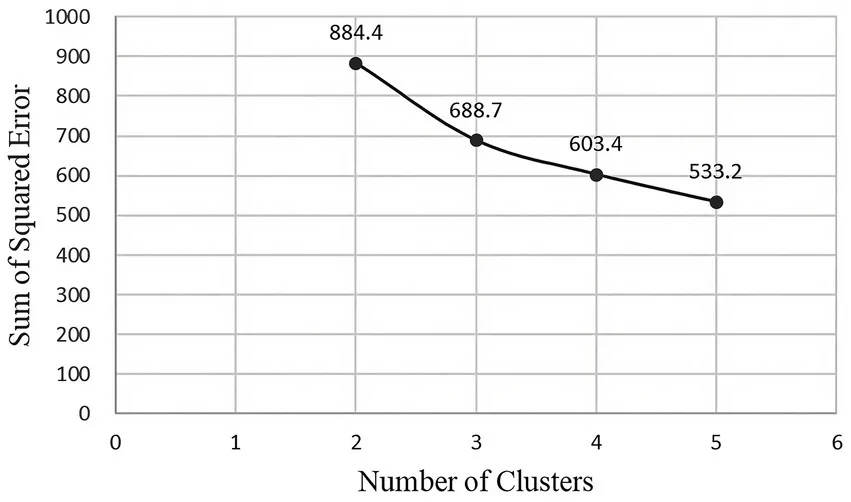
The sum of squares (SSE) elbow plot corresponding to different clustering numbers.

Considering the elbow rule, the convergence of the algorithm and the interpretability of the classification, four categories are finally determined as the optimal clustering scheme. There were significant differences in the four dimensions of disease treatment management (*F* = 204.98, *p* < 0.001), diet management (*F* = 225.72, *p* < 0.001), lifestyle management (*F* = 228.30, *p* < 0.001) and psychosocial management (*F* = 221.81, *p* < 0.001). Further pairwise comparison showed that the scores of C1 in each dimension were significantly higher than those of the other three categories, and C2 was significantly lower than the other three categories. There was no significant difference between C3 and C4 in the dimension of psychosocial management (*p* > 0.05), but there were significant differences in the other three dimensions (*p* < 0.05).

In order to verify the robustness of the clustering scheme, this study uses different random initial centers to repeat K-means clustering for three times. The results show that the sample size and clustering center of each category of the three clustering are highly consistent, indicating that the clustering scheme has good stability. Further, 70% of the samples (*n* = 279) were randomly selected for repeated clustering, and four categories with clear features were also identified. The distribution pattern of each category in the dimension was basically the same as that of the whole sample, which further supported the robustness of the clustering scheme.

### Characteristics and naming of potential categories of self-management behavior of gout patients

The *Z*-score distribution characteristics of the clustering centers of the four categories are shown in Figure 2. Based on the scores of the four dimensions and the level of knowledge, the self-management behavior of 402 patients with gout was divided into four potential categories.

**Figure 2:**
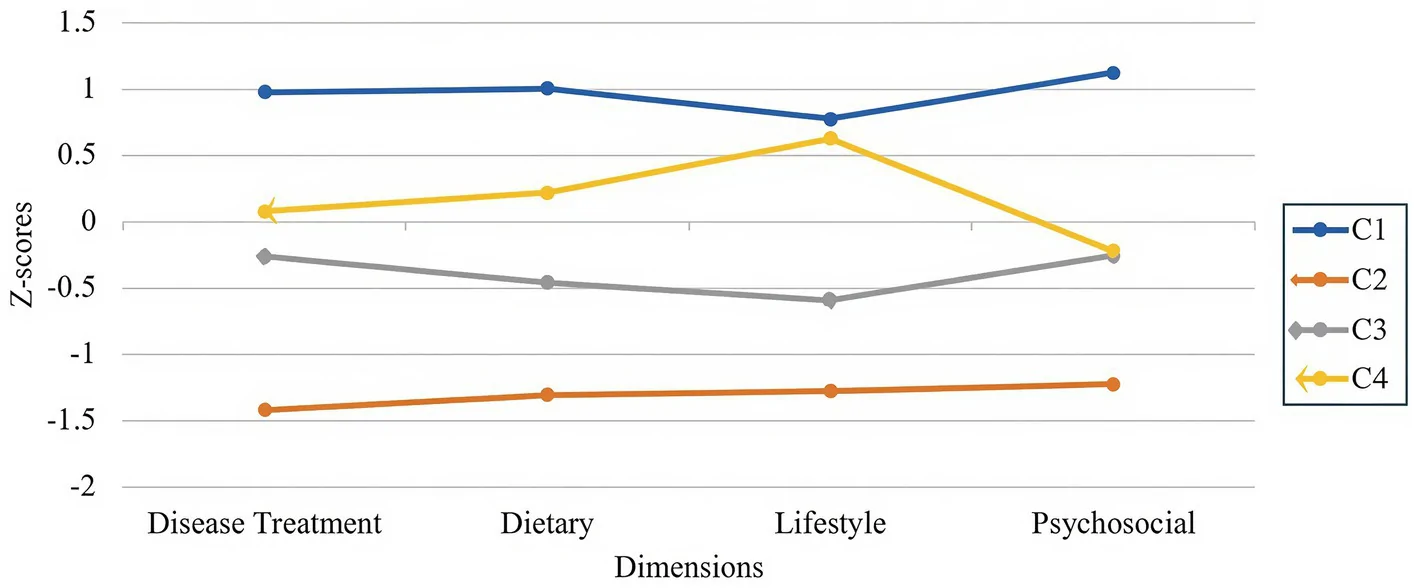
The distribution of dimension characteristics of self-management behavior of gout patients with different potential categories.

C1: The total score of self-management of these patients was the highest (176.06 ± 14.11), and the scores of the four dimensions were significantly higher than those of other categories. The level of knowledge was also the highest among the four groups (6.47 ± 1.87), with sufficient knowledge reserve and can be effectively transformed into comprehensive self-management behavior, and was named “high self-management-knowledge adequacy type.”

C2: The total score of self-management of these patients was the lowest (95.63 ± 13.90 points), the scores of the four dimensions were far lower than the average level, and the knowledge level was also the lowest among the four groups (4.81 ± 1.84 points). The knowledge reserve was seriously insufficient, the self-management was backward, and the risk of disease out of control was high, which was named “low self-management-knowledge deficiency type.”

C3: The total score of self-management of these patients was low (128.19 ± 8.68 points), the scores of the four dimensions were slightly lower than the average level, the level of knowledge was in the middle (5.81 ± 1.82 points), with a certain knowledge reserve but the efficiency of behavior transformation was insufficient, which was named “moderate self-management-insufficient transformation type.”

C4: The total score of self-management of these patients was higher (149.84 ± 9.69 points). The scores of the three dimensions of disease treatment management, diet management and lifestyle management were not lower than the average level. The psychosocial management dimension was slightly lower than the average level, and the knowledge level was low (5.27 ± 2.01 points). The behavior compliance was good but the knowledge reserve was insufficient, which was named “good self-management-knowledge deficiency type.”

### Comparison of knowledge level classification of gout patients with different potential categories of self-management behavior

The total scores of knowledge level of the four groups were: C1 (6.47 ± 1.87) points, C2 (4.81 ± 1.84) points, C3 (5.81 ± 1.82) points, C4 (5.27 ± 2.01) points. Kruskal–Wallis *H* test showed that there was a statistically significant difference in the level of disease knowledge among the four groups of gout patients (*H* = 35.314, *p* < 0.001, *η*^2^ = 0.081). The Bonferroni method was used for post-hoc pairwise comparisons. The results showed that the knowledge level of C1 was significantly higher than that of C2, C3 and C4 (*p* < 0.05). C2 was significantly lower than C1 and C3 (*p* < 0.05), but there was no significant difference with C4 (*p* = 0.785). There was no significant difference between C3 and C4 (*p* = 0.202). According to the questionnaire ≥ 7 points as the criterion of knowledge awareness, the knowledge level is divided into unknown (0 ~ 6 points) and known (≥7 points). Patients with high self-management-knowledge adequacy had the highest proportion of disease-related knowledge, while patients with low self-management-knowledge deficiency had the lowest proportion of disease-related knowledge, as shown in Table 1.

**Table 1:** Comparison of disease knowledge level classification of patients with different potential categories of self-management behavior.

Category	Number of samples	Unknown (0–6 points)	Awareness (≥7 points)
High self-management-knowledge adequacy	115	56(48.7%)	59(51.3%)
Low self-management-knowledge deficiency	62	50(80.6%)	12(19.4%)
Moderate self-management-insufficient transformation	124	81(65.3%)	43(34.7%)
Good self-management-knowledge deficiency	101	74(73.3%)	27(26.7%)

### Single factor analysis of gout patients with different potential categories of self-management behavior

Univariate analysis showed that patients with different self-management behaviors had statistically significant differences in disease knowledge, age, residence, working status, education level, tophi, number of acute flares in the past year and the latest SUA level (*p* < 0.05), as shown in Table 2.

**Table 2:** Single factor analysis of potential categories of self-management behavior in gout patients.

Variable (*n*, %)	High self-management-knowledge adequacy type (*n* = 115)	Low self-management-knowledge deficiency type (*n* = 62)	Moderate self-management-insufficient transformation type (*n* = 124)	Good self-management-knowledge deficiency type (*n* = 101)	*χ* ^2^ */H*	*P*
Gender					4.482	0.214
Male	113(98.3)	60(96.8)	122(98.4)	95(94.1)		
Female	2(1.7)	2(3.2)	2(1.6)	6(5.9)		
Age (years)					26.238	<0.001**
18 ~ 44	64(55.7)	26(41.9)	69(55.6)	26(25.7)		
45 ~ 59	26(22.6)	23(37.1)	41(33.1)	43(42.6)		
≥60	25(21.7)	13(21.0)	14(11.3)	32(31.7)		
BMI (kg/m^2^)					3.479	0.323
<18.5	1(0.9)	2(3.2)	3(2.4)	2(2.0)		
18.5 ~ 24.9	55(47.8)	27(43.5)	37(29.8)	44(43.6)		
25.0 ~ 29.9	46(40.0)	24(38.7)	51(41.1)	45(44.6)		
≥30.0	13(11.3)	9(14.5)	33(26.6)	10(9.9)		
Residence					25.748	<0.001**
Urban	102(88.7)	36(58.1)	88(71.0)	64(63.4)		
Rural	13(11.3)	26(41.9)	36(29.0)	37(36.6)		
Work status					9.346	0.025*
Work	86(74.8)	47(75.8)	104(83.9)	67(66.3)		
Jobless	29(25.2)	15(24.2)	20(16.1)	34(33.7)		
Educational level					14.059	0.003*
Middle school and below	32(27.8)	31(50.0)	55(44.4)	59(58.4)		
Senior middle school	19(16.5)	17(27.4)	25(20.2)	16(15.8)		
University and above	64(55.7)	14(22.6)	44(35.5)	26(25.7)		
Family per capita monthly income (yuan)					2.876	0.411
<3,000	13(11.3)	16(25.8)	19(15.3)	19(18.8)		
3,000 ~ 4,999	21(18.3)	17(27.4)	25(20.2)	22(21.8)		
5,000 ~ 9,000	52(45.2)	21(33.9)	56(45.2)	42(41.6)		
≥10,000	29(25.2)	8(12.9)	24(19.4)	18(17.8)		
Disease duration (years)					1.793	0.617
<1	4(3.5)	2(3.2)	2(1.6)	1(1.0)		
1 ~ 5	37(32.2)	18(29.0)	34(27.4)	25(24.8)		
6 ~ 10	49(42.6)	23(37.1)	52(41.9)	39(38.6)		
>10	25(21.7)	19(30.6)	36(29.0)	36(35.6)		
Family history					2.996	0.392
No	83(72.2)	50(80.6)	95(76.6)	82(81.2)		
Yes	32(27.8)	12(19.4)	29(23.4)	19(18.8)		
Tophi					8.674	0.034*
No	58(50.4)	21(33.9)	47(37.9)	33(32.7)		
Yes	57(49.6)	41(66.1)	77(62.1)	68(67.3)		
Disease stage					2.363	0.501
Acute stage	14(12.2)	18(29.0)	32(25.8)	18(17.8)		
Intercritical stage	54(47.0)	12(19.4)	30(24.2)	26(25.7)		
Chronic stage	47(40.9)	32(51.6)	62(50.0)	57(56.4)		
Number of acute flares in the past year (time)					22.416	<0.001**
0	30(26.1)	3(4.8)	14(11.3)	13(12.9)		
1 ~ 3	53(46.1)	16(25.8)	45(36.3)	48(47.5)		
>3	32(27.8)	43(69.4)	65(52.4)	40(39.6)		
SUA (μmol/L)					11.233	0.011*
<374	63(54.8)	15(24.2)	42(33.9)	39(38.6)		
374 ~ 452	31(27.0)	14(22.6)	17(13.7)	23(22.8)		
453 ~ 520	8(7.0)	11(17.7)	25(20.2)	15(14.9)		
>520	13(11.3)	22(35.5)	40(32.3)	24(23.8)		

*p < 0.05, **p < 0.001.

### Multivariate analysis of potential categories of self-management behavior in gout patients

Variables with statistical significance in univariate analysis were included in multivariate logistic regression analysis, and high self-management-knowledge adequacy (C1) was used as the reference group. The assignment of independent variables is shown in Table 3.

**Table 3:** Variable assignment.

Variable	Assignment
Age (years)	0 = 18 ~ 44; 1 = 45 ~ 59; 2 = ≥60
Residence	0 = urban; 1 = rural
Work status	0 = work; 1 = jobless
Educational level	0 = Middle school and below; 1 = Senior middle school; 2 = University and above
Tophi	0 = no; 1 = yes
Number of acute flares in the past year (time)	0 = 0; 1 = 1 ~ 3; 2= > 3
SUA (μmol/L)	0 = <374; 1 = 374 ~ 452; 2 = 453 ~ 520; 3= > 520
Disease knowledge score	Input with the original value

Multicollinearity was evaluated by variance inflation factor (VIF). The results showed that the VIF values of all independent variables were between 1.005 and 1.885, all less than 10, suggesting that there was no serious multicollinearity problem. The overall fitting of the model was good. The Nagelkerke *R*^2^ was 0.347, suggesting that the included independent variables could explain about 34.7% of the variation in the potential category of self-management behavior. The AIC and BIC values for the fitted model were 962.976 and 1154.805, respectively. The results showed that the contribution of disease knowledge score, age, residence, number of acute flares in the past year, and SUA level to the model was statistically significant (*p* < 0.05). The specific parameter estimation of each variable is shown in Table 4.

**Table 4:** Multivariate logistic regression analysis of the potential categories of self-management behavior of gout patients.

Variable	*β*	SE	Wald *χ*^2^	*P*	OR	95% CI
C2 vs. C1^a^						
Disease knowledge score	−0.474	0.106	20.001	<0.001**	0.6227	0.506 ~ 0.765
Residence (urban vs. rural)	−1.316	0.435	9.148	0.002*	0.268	0.114 ~ 0.629
Number of acute flares in the past year (>3 vs. 0 time)	2.060	0.709	8.440	0.004*	7.845	1.955 ~ 31.489
SUA (374 ~ 452 vs. <374 μmol/L)	1.133	0.486	5.427	0.020*	3.103	1.197 ~ 8.047
SUA (453 ~ 520 vs. <374 μmol/L)	1.783	0.605	8.688	0.003*	5.949	1.817 ~ 19.470
SUA (>520 vs. <374 μmol/L)	2.021	0.503	16.155	<0.001**	7.544	2.816 ~ 20.208
C3 vs. C1^a^						
Disease knowledge score	−0.220	0.085	6.742	0.009*	0.802	0.680 ~ 0.947
Residence (urban vs. rural)	−0.857	0.383	5.004	0.025*	0.424	0.200 ~ 0.899
Number of acute flares in the past year (>3 times vs. 0 times)	1.125	0.439	6.584	0.010*	3.081	1.304 ~ 7.277
SUA (453 ~ 520 vs. <374 μmol/L)	1.566	0.484	10.454	0.001*	4.789	1.853 ~ 12.378
SUA (>520 vs. <374 μmol/L)	1.452	0.400	13.209	<0.001**	4.274	1.953 ~ 9.353
C4 vs. C1^a^						
Disease knowledge score	−0.280	0.088	10.095	0.001*	0.755	0.635 ~ 0.898
Age (45–59 vs. 18–44 years)	1.055	0.368	8.199	0.004*	2.872	1.395 ~ 5.912
Residence (urban vs. rural)	−1.194	0.388	9.464	0.002*	0.303	0.142 ~ 0.648
SUA (>520 vs. <374 μmol/L)	1.091	0.433	6.357	0.012*	2.978	1.275 ~ 6.955

^a^ is the reference group; *p < 0.05, **p < 0.001.

## Discussion

### There are deficiencies in self-management behavior and disease knowledge level of gout patients

The results of this study showed that the score of self-management assessment scale for gout patients was (142.30 ± 29.53), which was above the average level, which was consistent with the previous research conclusions (Hao and Wang, 2025). From the overall mean point of view, the score of psychosocial management dimension is relatively low (20.88 ± 5.89 points), which is the weak link in the self-management of gout patients. The results of cluster analysis showed that this weakness was mainly concentrated in specific patient subgroups. The scores of psychosocial management in patients with C2 (low self-management-knowledge deficiency), C3 (moderate self-management-insufficient transformation) and C4 (good self-management-knowledge deficiency) were lower than the average level, while the scores of this dimension in patients with C1 (high self-management-knowledge adequacy) were significantly higher than the average, suggesting that psychosocial management problems were different among different categories of patients and had group heterogeneity. Qualitative research also found that gout patients had psychological self-management disorders such as passive negative coping, self-deception and stigma (Hao et al., 2024), and patients with lower levels of psychological capital had worse self-management behaviors (Wang Y. et al., 2025). Therefore, psychosocial factors are an important breakthrough in improving the self-management behavior of gout patients, which need to be focused on in clinical intervention.

In addition to the weak psychosocial management, the level of disease knowledge of gout patients is also not optimistic. In this study, the knowledge level of gout patients was (5.71 ± 1.97), and the awareness rate was only 35.1%, which was at a low level. The lack of e-health literacy may hinder the effective acquisition of knowledge and behavioral transformation (Qian et al., 2024), while gout patients generally have limited knowledge and negative attitudes (Yan et al., 2026). The above results suggest that there is still a gap between the coverage of current gout health education and the actual mastery of patients, and it may be difficult to meet the knowledge needs of patients by relying solely on routine education. Therefore, it is necessary to explore a more systematic and continuous health education model and carry out stratified intervention for people with different characteristics.

### Disease knowledge level emerged as the core feature to distinguish the potential categories of self-management behavior

In this study, four self-management behavior subgroups with significant differences were identified by K-means clustering analysis. The knowledge scores of the four groups showed a gradient distribution of C1 (6.47 points) > C3 (5.81 points) > C4 (5.27 points) > C2 (4.81 points), which was highly consistent with the level of self-management behavior, and the difference between the groups was statistically significant (*p* < 0.001). Multivariate logistic regression revealed that disease knowledge score was significantly associated with the self-management behavior categories.

According to the theoretical model of knowledge, belief and behavior, knowledge needs to be transformed by attitude to drive the change of behavior (Wang et al., 2025). The accumulation of knowledge is not automatically equivalent to the change of behavior. In this study, C3 patients had higher knowledge scores than C4 patients, but lower self-management total scores, suggesting that the core problem of C3 is the insufficient efficiency of translating knowledge into behavior, rather than a lack of knowledge itself. This phenomenon is known as the “knowledge–behavior gap.” Attitude plays a key role as a bridge between knowledge and practice (Shi et al., 2026). If the patient’s cognition of the disease only stays at the “know” level and fails to translate into positive health beliefs, behavioral changes are difficult to occur.

Of note, C4 exhibited a pattern opposite to that of C3. Their knowledge scores were relatively low, yet their self-management scores remained high. This pattern suggests that knowledge may not be the sole driver of behavior change, as behaviors can also be prompted by external cues or sustained by established routines. Patients with a lack of psychological capital often lack the intrinsic motivation and confidence to put knowledge into action (Wang Y. et al., 2025).

The contrasting patterns of C3 and C4 can be further understood through the Health Belief Model (HBM; Rosenstock et al., 1988). In the HBM framework, health behaviors are shaped by perceived susceptibility, perceived severity, perceived benefits, perceived barriers, cues to action, and self-efficacy, with knowledge functioning as a modifying factor that influences these perceptions. For C3 patients, who demonstrated adequate knowledge but low behavioral performance, this pattern is consistent with the possibility that perceived barriers to self-management or low perceived benefits of behavioral change may outweigh their knowledge. For C4 patients, their good behavioral adherence despite low knowledge aligns with the role of strong external cues to action or established routines, which may sustain behavior even when internal knowledge is limited.

Collectively, C3 and C4 represent two contrasting patterns of knowledge–behavior mismatch: C3 reflects “knowing but not doing,” while C4 reflects “doing but not knowing.” The coexistence of these two patterns suggests that clinical interventions should be tailored to address different types of knowledge–behavior gaps. For C3, interventions should focus on attitude change and behavioral activation; for C4, efforts should maintain existing behaviors while supplementing knowledge and assessing the sustainability of external support systems.

### Factors associated with self-management behavior categories in gout patients

Multivariate analysis showed that age, residence, number of acute flares in the past year, and SUA level were significantly associated with the self-management behavior category.

In terms of age, the risk of belonging to C4 in patients aged 45–59 years was 2.872 times higher than that in patients aged 18–44 years (OR = 2.872, 95% CI: 1.395–5.912), suggesting that although middle-aged patients had good self-management behavior compliance, their disease knowledge reserve was relatively insufficient. However, the phenomenon of non-compliance with uric acid lowering therapy is more common in young patients (Uhlig et al., 2025), and the older patients have better medication compliance (Schulz et al., 2024). Patients of different ages face different management dilemmas. Young patients focus on improving behavioral compliance and strengthening long-term management awareness, while middle-aged patients need to strengthen disease knowledge education on the basis of maintaining existing behavior.

The risk of belonging to C2, C3 and C4 in urban patients was significantly lower than that in rural patients (C2 vs. C1: OR = 0.268, 95% CI: 0.114–0.629; C3 vs. C1: OR = 0.424, 95% CI: 0.200–0.899; C4 vs. C1: OR = 0.303, 95% CI: 0.142–0.648). Previous studies have found that there are differences in disease education preferences between urban and rural gout patients, suggesting that health education needs to adopt differentiated strategies (Treharne et al., 2018). However, studies have also pointed out that the urban–rural gap has a smaller role in gout treatment compliance (Sutton et al., 2023). In spite of this, rural gout patients still face greater difficulties in self-management due to the relative lack of medical resources and limited coverage of health education, and should be the focus of health intervention.

The risk of belonging to C2 in patients with acute flares > 3 times was 7.845 times higher than that in patients without acute flares (OR = 7.845, 95% CI: 1.955–31.489). The risk of C3 was 3.081 times higher than that of patients with 0 acute flares (OR = 3.081, 95% CI: 1.304–7.277). Frequent acute episodes of gout may weaken patients’ self-management confidence and sense of disease control, and make them fall into a vicious circle of “attack-behavior slack-recurrence “(Hao and Wang, 2025). It is suggested that attention should be paid to patients with frequent acute flares, and their self-management behavior should be evaluated in time and targeted intervention should be given.

The risk of belonging to C2, C3 and C4 was significantly increased in patients with SUA > 520 μmol/L (C2 vs. C1: OR = 7.544, 95% CI: 2.816–20.208; C3 vs. C1: OR = 4.274, 95% CI: 1.953–9.353; C4 vs. C1: OR = 2.978, 95% CI: 1.275–6.955). SUA compliance is the core goal of gout treatment, and the positive feedback brought by SUA control can strengthen patients’ behavioral maintenance motivation. Thematic nursing education can enhance the patient’s disease awareness, control SUA levels, reduce acute episodes (Gao and Meng, 2025), and awareness of SUA lowering treatment goals and the need for long-term medication is also associated with good treatment compliance (Tang et al., 2026). Knowledge improvement may facilitate better SUA control, and compliance is a key link in transforming knowledge into clinical benefits.

### Targeted intervention strategies for the potential categories of self-management behavior of gout patients

The four potential categories identified in this study have different characteristics and intervention needs, suggesting that stratified precise intervention should be implemented in clinical nursing.

With maintenance and consolidation as the core, C1 maintains its good behavior habits through regular follow-up and positive feedback to prevent behavioral laxity due to the disease entering a stable period; at the same time, it can play a peer demonstration effect to improve the behavior of other types of patients. C2 was the group with the highest health risk in the four categories. The awareness rate was only 19.4%, and the number of acute flares > 3 times accounted for 69.4%. Intervention should take basic disease knowledge education as the core, using easy-to-understand publicity methods, combined with behavioral contracts and progressive goals to help patients establish behavioral habits. Thematic nursing education is superior to traditional health education in enhancing disease cognition (Gao and Meng, 2025). Mobile health model can expand the accessibility and sustainability of health education (Wang Y. et al., 2024). Structured medication process helps to promote patients’ long-term medication compliance (Crum et al., 2024). The core issue of C3 is the “knowledge gap,” and intervention should focus on attitude change. Knowledge needs to be driven by attitude to change behavior (Wang D. et al., 2024). Health beliefs are strengthened through case teaching and scenario simulation. At the same time, peer support education is used to improve patients’ self-efficacy, and family members are included in the support system to help supervise and encourage patients’ daily behavior management. The behavioral improvement of C4 may be more dependent on external factors than internal knowledge. Education on disease knowledge should be strengthened on the basis of maintaining existing behaviors to help patients understand the necessity and long-term benefits of healthy behaviors. Gout patients have diverse needs for disease management support, suggesting that external support plays an important role in behavior maintenance (van der Ven et al., 2024). At the same time, it is necessary to pay attention to the psychological state of patients, timely identify emotional problems such as anxiety and depression, and recommend them to seek professional psychological support if necessary (Do et al., 2025).

## Conclusion

Through K-means cluster analysis, the self-management behavior of gout patients was divided into four potential categories: high self-management-knowledge adequacy type, low self-management-knowledge deficiency type, moderate self-management-insufficient transformation type and good self-management-knowledge deficiency type. Disease knowledge score emerged as a distinguishing characteristic among the four self-management subgroups. Patients in the high self-management-knowledge adequacy group had higher knowledge scores, whereas those in the low self-management-knowledge deficiency group had the lowest scores. These findings highlight the importance of disease knowledge as a distinguishing characteristic of different self-management subgroups. There are differences in the level of disease knowledge among different categories of patients, suggesting that the level of knowledge is an important factor in distinguishing different categories. Urban residence, number of acute flares in the past year ≤ 3 times, good control of SUA level (<374 μmol/L), and age of 18–44 years were associated with a higher likelihood of belonging to the high self-management-knowledge adequacy type. Medical staff should carry out stratified precise intervention according to different types of characteristics, focusing on high-risk groups in rural areas, those with frequent acute flares, and those with low disease knowledge.

Although this study has limitations such as single-center sampling, cross-sectional design, and information bias, the results still have certain clinical practice significance, which can provide reference for future multi-center, large-sample longitudinal studies and further improve the precise health management model for gout patients. In addition, the convenience sample from a single tertiary hospital limits the generalizability to community-dwelling gout patients. Future studies can use longitudinal design to track the dynamic changes of patients in various categories and explore the effects of intervention programs based on potential categories to verify the effectiveness of the precision strategy proposed in this study.

## Data Availability

The original contributions presented in the study are included in the article/supplementary material, further inquiries can be directed to the corresponding author.
